# High Sensitivity Troponin T as Complementary Modality for Determining Doxorubicin Regimen Cardiotoxicity in Non-Hodgkin Lymphoma Patients

**DOI:** 10.34172/apb.2022.017

**Published:** 2020-10-19

**Authors:** Ami Ashariati Prayogo, Satriyo Dwi Suryantoro, Merlyna Savitri, Winona May Hendrata, Andi Yasmin Wijaya, Budi Susetyo Pikir

**Affiliations:** ^1^Division of Hematology-Oncology, Department of Internal Medicine, Dr. Soetomo Teaching Hospital, Surabaya, East Java, Indonesia.; ^2^Department of Internal Medicine, Airlangga University Hospital, Surabaya, East Java, Indonesia.; ^3^Faculty of Medicine, Airlangga University, Surabaya, East Java, Indonesia.; ^4^Department of Cardiology and Vascular Medicine, Dr. Soetomo Teaching Hospital, Surabaya, East Java, Indonesia.

**Keywords:** Cardiotoxicity, Doxorubicin, Left ventricular function, Non-Hodgkin lymphoma, Troponin T, Cancer

## Abstract

***Purpose:*** This study aims to evaluate the role of high-sensitivity troponin T (hsTnT) as a complementary tool for determining cardiotoxicity in non-Hodgkin lymphoma (NHL) patients receiving cyclophosphamide, doxorubicin, vincristine and prednisone (CHOP) regimen chemotherapy.

***Methods:*** We included 35 patients diagnosed with NHL who received CHOP chemotherapy. Left ventricular ejection fraction (LVEF) and hsTnT were measured at two time points: before the first cycle (pre-test) and after the fourth cycle (post-test). The LVEF and hsTnT were analysed using IBM SPSS version 24 through the paired-sample T-test, Wilcoxon signed-rank test, Pearson’s correlation and Spearman’s correlation.

***Results:*** There was a significant difference in both LVEF and hsTnT between pre-chemotherapy and post-4th chemotherapy cycles (*P* = 0.001). However, more contrast difference from the baseline value of hsTnT compared to LVEF could be observed. LVEF did not detect any deterioration in myocardial function. However, 10 out of 35 subjects exhibit hsTnT higher than the 99th percentile of the population (>14 pg/ml), suggesting that myocardial injury (MI) could be detected. There was no correlation between LVEF and hsTnT (*P* > 0.05).

***Conclusion:*** HsTnT, together with LVEF, could complement each other and offer better coverage for detecting cardiotoxicity during the administration of CHOP in NHL patients. An insignificant correlation between hsTnT and LVEF showed that cardiotoxicity existed in a broad spectrum including cellular damage and functional impairment, as hsTnT represents cellular damage, and LVEF reflects heart functional capacity.

## Introduction

Non-Hodgkin lymphoma (NHL) accounts for 90% of lymphoma cases around the world, and 509 590 new cases of NHL were recorded all over the world in 2018.^1^ In the clinical setting, NHL presents with a wide range of clinical manifestations that makes treating NHL a challenge among clinicians.^2^ Chemotherapy is the treatment of choice for NHL.^3^ The CHOP (cyclophosphamide, doxorubicin, vincristine and prednisone) regimen as combined chemotherapy is the first-line regimen for the treatment of NHL, even though its use is limited due to doxorubicin cardiotoxicity.^4-6^ Heart failure (HF) as manifestation of chemotherapy-associated cardiotoxicity increases mortality and morbidity of patients receiving anthracycline, even after the end of chemotherapy.^7,8^

Left ventricular ejection fraction (LVEF) measurement by echocardiography is a primary cardiotoxicity monitoring modality. A decrease in LVEF reflects the deterioration of heart contractility as a manifestation of doxorubicin cardiotoxicity.^8^ However, regardless of echocardiography benefits, the heart condition that is represented in LVEF is limited to cardiac functional spectrum. Prior to functional impairment, damage at the cellular level might have occurred.

Transient changes at the cellular level might go undetected due to the absence of clinical signs and symptoms, along with normal functional parameters. Cardiac marker level is currently an essential modality for diagnosing myocardial infarction as a representation of cardiac muscle destruction.^9^ High-sensitivity troponin T (hsTnT) is a measurement of the troponin T which released from destroyed cardiomyocytes with adequate sensitivity.^10^ HsTnT might be utilised as a complementary modality in the determination of doxorubicin cardiotoxicity, covering the cellular spectrum of cardiotoxicity. Therefore, this study aimed to evaluate role of hsTnT in detecting cardiotoxicity in NHL patients receiving the CHOP chemotherapy regimen, given that it has never been assessed before, especially in the Indonesian population.

## Materials and Methods

The subjects of this research were all NHL patients who were admitted to the haematology-medical oncology ward of the Dr. Soetomo Teaching Hospital, Surabaya, East Java, Indonesia, from August to December 2016. The inclusion criteria were: patients above 18 years and patients who were on CHOP chemotherapy. The exclusion criteria were: history of myocardial infarction, diabetes mellitus, chronic kidney disease, liver cirrhosis and having undergone chemotherapy and radiotherapy sessions before this study was conducted. All participants signed inform consent forms before they were recruited into the study.

All chemotherapy procedures were conducted in the Internal Medicine Department of the Dr. Soetomo Teaching Hospital, Surabaya, East Java, Indonesia. The combination of CHOP chemotherapy regimen consists of 750 mg/m^2^ intravenous cyclophosphamide, 50 mg/m^2^ intravenous doxorubicin, 1.4 mg/m^2^ intravenous vincristine and 100 mg oral prednisone from day one until five^11^ One cycle of chemotherapy was performed every three weeks.

Echocardiography was performed for all the subjects before the first chemotherapy cycle and after the fourth cycle. A trained practitioner performed LVEF measurement by two-dimensional echocardiography. Blood samples were obtained from the patients at two time points: before starting chemotherapy and after the fourth chemotherapy cycle. The serum hsTnT levels from blood samples was measured through electro-chemiluminescent methods, using the electrochemiluminescence immunoassay analyser (ECLIA) (Roche Cobas E602 multi-channel analyser). HsTnT would be marked as myocardial injury (MI) if the level is higher than its 99^th^ percentile (> 14 pg/mL).^9,12^

Both results from LVEF and hsTnT were tested for normality using the Shapiro-Wilk test. LVEF percentage and hsTnT level before and after chemotherapy were compared using the Wilcoxon signed-rank test for whole subjects and the paired-sample T-test for MI subjects. Spearman’s correlation was used to assess the correlation between LVEF and hsTnT for all the subjects. Pearson’s correlation was used for assessing LVEF and hsTnT in MI patients. Data were presented using graphs as per the recommendation for non-parametric data presentation with modification.^13^

## Results

### Subject characteristics

**Figure 1:**
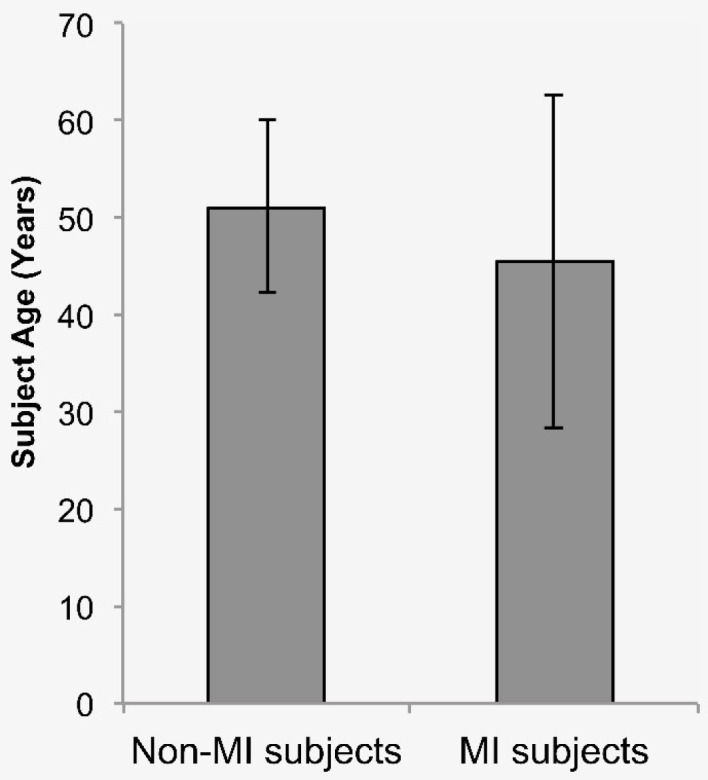
Age difference between patient who developed MI and without MI.

Thirty-five patients met the eligibility criteria for the study. The ages of the patients ranged from 20–71 years. All the subjects provided their written informed consent prior to their enrolment into the study. Up to the fourth cycle, the total cumulative dose of doxorubicin received by every subject was 200 mg/m^2^. During this study, no cardiac failure or other cardiovascular event occurred. Before the start of chemotherapy, all subjects had serum hsTnT levels below 14 pg/mL. MI occurred in 10 patients after the 4^th^ chemotherapy cycle (Table 1). Analysis of the difference in age between subjects who developed MI (*n* = 10; 45.40 ± 17.18) and subjects who did not develop MI (*n* = 25; 51.16 ± 8.86) showed no significant difference (*P*= 0.333) (Figure 1).

**Table 1:** Patient Characteristics

**Characteristic**	***n***	**Percent**
Gender		
Male	25	71.42
Female	10	28.58
Age
< 60 years old	25	71.42
≥ 60 years old	10	28.58
NHL clinical stage		
I	7	20
II	7	20
III	20	57
IV	1	2.86
NHL histopathological classification		
Diffuse large B cell, high grade	20	57.14
Diffuse large B cell, intermediate grade	10	28.57
Small B cell lymphoma	3	8.57
Follicular lymphoma	2	5.72
HsTnT before chemotherapy		
< 14 pg/mL (non-MI)	35	100
≥ 14pg/mL (MI)	0	0
HsTnT after chemotherapy
< 14 pg/mL (non-MI)	25	71.42
≥ 14pg/mL (MI)	10	28.58

We analyzed the difference of age between subjects who developed MI (n = 10, 45,40 ± 17,18) and subjects without MI (n = 25, 51.16 ± 8,86). The result showed no significant difference (*P* = 0.333).

### Left ventricular ejection fraction

**Figure 2:**
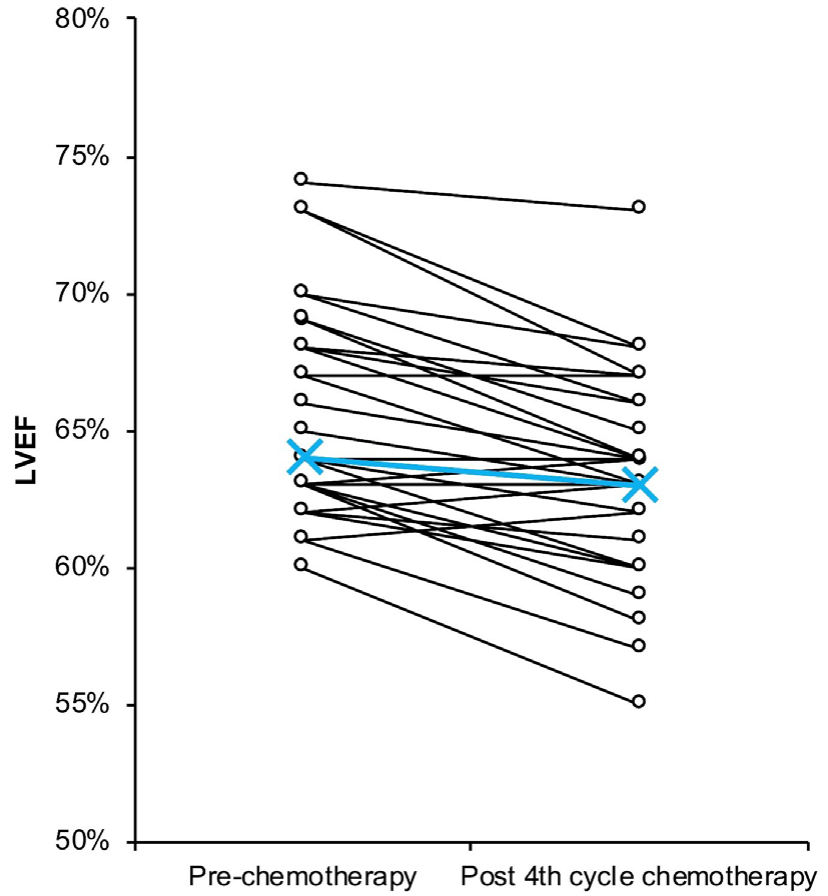
LVEF change from pre-chemotherapy to post 4th cycle chemotherapy. Blue line and cross shows LVEF median.

Echocardiography measurement before the first CHOP cycle showed that there were no subjects with LVEF under 50% (median = 64%; range = 60%–74%)^14^ (Figure 2). There was a significant decrease in LVEF after the fourth cycle showed based on the Wilcoxon signed-rank test (median = 63%; range = 55%–73%; *P*< 0.001). Based on paired-sample *t*test on MI subjects, there was a significant decrease in LVEF after 4^th^ cycle of chemotherapy (*P*< 0.001) (Table 2).

**Table 2:** Pre and post 4^th^ chemotherapy cycle LVEF and hsTnT comparison

	**Pre-chemotherapy**	**Post-4** ^th^ ** chemotherapy cycle**	***P***
All subjects (n = 35)			
LVEF (%)	64 (60-74)^a^	63 (55-73)^a^	0.001^c^
hsTnT (pg/mL)	5.1 (3-11.96)^a^	10.1 (3.77-31.15)^a^	0.001^c^
Cardiac parameter changes	2 (1-6)^a^	4.4 (0.3-19.38)^a^	0.001^c^
Subjects with MI (n = 10)
LVEF (%)	65.7 ± 3.50^b^	62.4 ± 3.72^b^	0.001^d^
hsTnT (pg/mL)	9.13 ± 2.64^b^	21.08 ± 4.85^b^	0.001^d^
Cardiac parameter changes	3.34 ± 1.57^b^	11.95 ± 5.70^b^	0.001^d^

^a^ The results are described as median (range). ^b^ The results are described as mean ± standard deviation (SD). ^c^ *P*value is reported based on the analysis of Wilcoxon signed rank test (95% CI). ^d^ *P*value is reported based on the analysis of paired sample *t*test (95% CI).

### High-sensitivity troponin T

**Figure 3:**
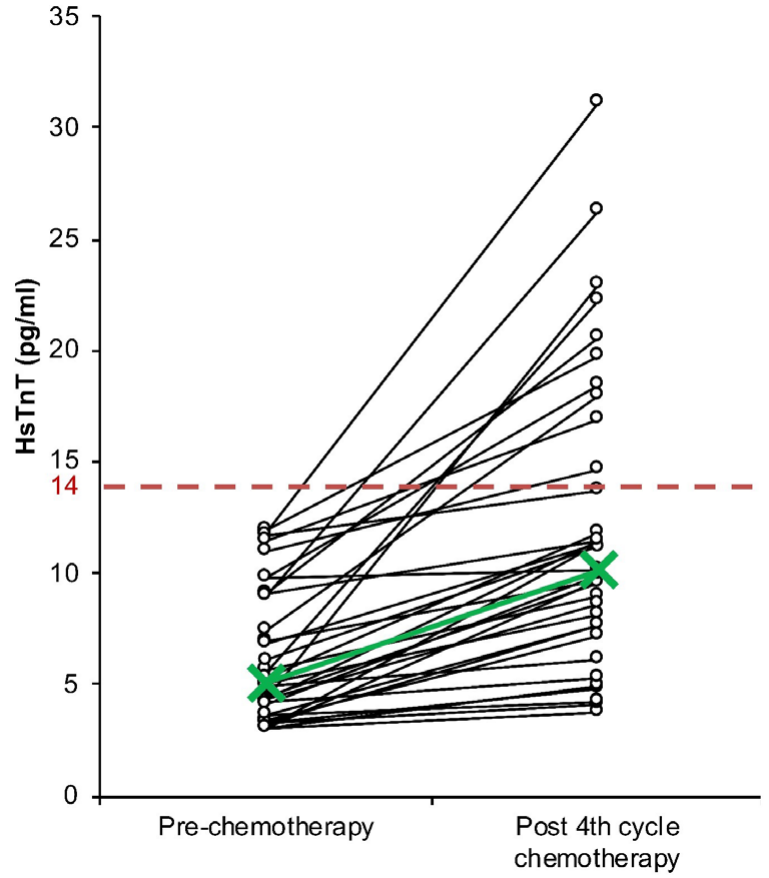
HsTnT change from pre-chemotherapy to post 4th cycle chemotherapy. Green line and cross shows HsTnT median of each time point. Red dashed line shows 99th percentile value at 14 pg/mL.

HsTnT values of all subjects before the first cycle were lower than 14 pg/mL, the 99^th^ percentile (median = 5.1 pg/mL; range = 3.0–11.96 pg/mL)^12^ (Figure 3). After the fourth chemotherapy cycle, hsTnT was significantly higher based on the Wilcoxon signed-rank test (median = 10.1 pg/mL; range = 0.3–19.38 pg/mL; *P<*0.001) (Table 2). Furthermore, even when the median hsTnT level after the fourth chemotherapy cycle is under 14 pg/mL, there were 10 out of 35 subjects that had hsTnT values higher than 14 pg/mL (Figure 3). Based on the paired-sample *t* test for MI subjects, there was a significant increase in hsTnT (*P*< 0.001). Evaluation of the difference between LVEF and hsTnT changes across all subjects and MI subjects showed a significant difference (*P*< 0.001) (Table 2).

### LVEF and hsTnT correlation

The correlation between LVEF and hsTnT in all subjects at the same time point was tested using Spearman’s correlation analysis. The test showed that there was no significant correlation between LVEF and hsTnT value at pre-chemotherapy (*P*= 0.652) and post-4^th^ cycle chemotherapy (*P* = 0.316). There was also no significant correlation between LVEF and hsTnT changes (*P*= 0.117) (Table 3).

**Table 3:** Pre and post 4^th^ chemotherapy cycle LVEF and hsTnT correlation

**Samples**	***P***
All subjects (n = 35)	
Pre-chemotherapy	0.652^a^
Post 4^th^ cycle chemotherapy	0.420^a^
Cardiac parameter changes	0.052^a^
Subjects with MI (*n* = 10)	
Pre-chemotherapy	0.068^b^
Post 4^th^ cycle chemotherapy	0.722^b^
Cardiac parameter changes	0.820^b^

^a^ *P*value is reported based on the analysis of Spearman’s correlation test (95% CI). ^b^ *P*value is reported based on the analysis of Pearson’s correlation test (95% CI).

Pearson’s correlation analysis was used to assess LVEF and hsTnT correlation in subjects with MI (*n* = 10). We observed that there was no correlation between LVEF and hsTnT at pre-chemotherapy (*P*= 0.068) and post-4^th^ cycle chemotherapy (*P*= 0.722). There was also no significant correlation between changes in LVEF and hsTnT (*P*= 0.082) (Table 3).

## Discussion

The advantage of echocardiography lies in its non-invasiveness and flexibility in viewing the heart’s structure, not to mention its wide availability in medical facilities. LVEF measurement until now retains several factors that potentially affect the reliability of the result, mostly due to its operator-dependent nature.^15^ As LVEF depends on both cardiac preload and afterload, this measurement is commonly used for assessing the clinical outcome of patients with HF.^16^ However, HF with certain risk factors presents with preserved LVEF.^17^ A significant reduction of LVEF at the end of chemotherapy cycles was shown to be a potential predictive factor for cardiotoxicity.^18^ Current guidelines on chemotherapy-associated cardiotoxicity by the European Society of Medical Oncology suggests periodic LVEF measurement to detect cardiotoxicity. Withholding of a suspected chemotherapeutic agent is recommended if there are symptoms of HF, a decrease of LVEF > 20%, or a decrease of LVEF > 10% with total LVEF higher than 50%.^19^

All the 35 participants in this study did not show any symptom of HF from the start of chemotherapy to after the fourth cycle of chemotherapy. LVEF measurement showed an overall decrease after the fourth cycle of chemotherapy (*P*= 0.001) (Figure 2, Table 2). Normal LVEF varies between 50% until 70%. There was no subject having LVEF under 50% prior to chemotherapy. After the fourth cycle, there was no subject with an LVEF decrease of more than 10% and no subject in whom LVEF fell below 50% (Figure 2). The decrease in LVEF in every subject might be a manifestation of clinically-covert functional impairment. The median of difference in our study was 2%. However, a decrease in LVEF was observed in most of the subjects, asserting minor LVEF change did occur. Previous studies demonstrated that the level of reduction in LVEF during chemotherapy varies, and a significant reduction could serve as a predictor for future HF.^18^ There are possibly other factors that could create bias into LVEF measurement including other medication taken during the study, which we did not assess in our study.

Circulating troponins as a cardiac marker is an essential diagnostic modality for the detection of myocardial infarction.^9^ HsTnT is currently preferable due to standardised antibody manufacturing and decent sensitivity with a lower limit of detection.^10,20^ HsTnT uses a modified cardiac troponin T (cTnT) detection antibody to increase its specificity and decrease cross-reaction with skeletal muscle troponin T, cardiac troponin I, skeletal muscle troponin I and troponin C.^20^ MI is defined as the elevation of cardiac troponin above the 99^th^ percentile in the population.^9^ The normal cut-off point for hsTnT in the peripheral circulation is defined based on the 99^th^ percentile of the population (14 pg/mL in our case).^12^ This method has a low limit of detection, which could represent an early warning sign for clinicians of cardiac muscle damage before progressing further into clinical symptoms.^10^ The absence of signs and symptoms of HF does not rule out MI.^21^

Our study revealed that all subject did not show any symptoms of HF from the start of chemotherapy to after the 4^th^ cycle of chemotherapy. There was, however, a significant increase in overall hsTnT (Table 2). Elevation of hsTnT is a sign of ongoing damage in myocardial cells. Moreover, 10 out of the 35 subjects developed MI (hsTnT > 14 pg/mL) (Figure 2). Our study had excluded patients with history of cardiac events, diabetes mellitus and chronic kidney disease, all of which could cause hsTnT elevation.^22,23^ Yet, our study did not impose an upper limit for patient age, in which older patient were at increased risk of cardiac events. However, there was no significant difference in age between subjects who developed MI and those who did not (Figure 1). Cardiac marker levels are also affected by time after injury. Also, our study did not uniformise the moment of taking blood samples after the 4^th^ cycle of chemotherapy.

Up to the fourth cycle, the subjects of this study equally received a 200 mg/m^2^ cumulative dose of doxorubicin. The incidence of doxorubicin-induced cardiotoxicity varies with its cumulative dose. The current recommended maximum cumulative dose for doxorubicin is 400–450 mg/m^2^.^24^ A cohort study involving patients treated with doxorubicin ranging from one cycle to more than eight cycles revealed that 4% of the subjects develop HF, with a significantly higher risk in higher cycle counts.^7^ Another study showed that breast cancer patients receiving < 300 mg/m^2^ doxorubicin presented with subclinical cardiomyopathy through echocardiography examination.^25^ Cardiac magnetic resonance imaging in breast cancer patients receiving a 240 mg/m^2^ cumulative dose of doxorubicin revealed left ventricular atrophy, without reduced LVEF or clinical symptoms, suggesting that clinically-covert cardiotoxicity had occurred at a cumulative dose of doxorubicin below 500 mg/m^2^.^26^

Our study did not find any significant correlation between LVEF and hsTnT (Table 3). We carried out a separate analysis for the 10 subjects with MI in order to obtain a keener analysis of the correlation of LVEF change in patients with MI. The result showed that there was no significant correlation between the differences in hsTnT and LVEF (Table 3). The absence of a correlation indicates that the cardiotoxicity effect shown by both of the assessments lies on a different spectrum, suggesting that both tests should be used in a complementary manner.

Prior to functional impairment, damage at the cellular level might have occurred. Damage at the cellular level can progress to affecting the organ function as a whole. Transient change at the cellular level might go undetected due to the absence of signs and symptoms, along with normal functional parameters. Cardiac marker levels are currently an essential modality for diagnosing myocardial infarction.^9^ Given the cellular pathophysiology of anthracycline cardiotoxicity, injury at the cellular level should be detectable, and potentially serve as an early detection method complementary to LVEF measurement.

There are few mechanisms proposed for explaining doxorubicin cardiotoxicity: damage by oxidative stress, alteration resulting in cardiac contractility impairment and induction of apoptosis.^27,28^ Oxidative stress is caused by anthracycline uptake of the myocardial cells and causing increased activity of the superoxide cleaving enzyme.^29^ A build-up of oxidant concentration within cells also induces apoptosis response through mitochondrial membrane protein.^29^ A radiopharmaceutical marker study has demonstrated an elevation of apoptotic markers in mice before reduced ejection fraction were detectable through echocardiography.^30^ The measurement of cellular markers is, thus, an on-point method of assessing the effect of doxorubicin toward myocardial cells, but not as a substitute for LVEF measurement.

The heart function and integrity as an organ could be assessed using various modalities, with each representing a different element of the heart. The cardiac functional aspect as a blood-pumping organ could be measured using echocardiography in the form of LVEF.^16^ The measurement of LVEF through echocardiography mainly demonstrates cardiac function, whereas hsTnT demonstrates cardiac injury at the cellular level.^21^ Whether these two tests should be used in complementarity to one another or even simultaneously in every patient is one of the questions this study would like to raise.

## Conclusion

This study suggests that in the clinical setting, cardiotoxicity of chemotherapy can manifest in a wide spectrum, from cellular level injury to functional incapacity. In the context of doxorubicin cardiotoxicity, LVEF and hsTnT are not substitutes for one another, as both result did not correlate. Rather, the measurement of hsTnT or other cardiac markers should be studied further as a routine procedure for patients receiving doxorubicin chemotherapy as a potential prognostic marker for cardiotoxicity. Both LVEF and hsTnT are recommended for the detection of cardiotoxicity due to doxorubicin at a dose of 200 mg/m^2^ in patients with NHL.

This study was carried out over a limited period, resulting in a limited number of participants. Further studies should be conducted with more systematic sampling methods.

## Ethical Issues

This study was designed following the Declaration of Helsinki and had been ethically approved by the Dr. Soetomo Teaching Hospital Ethical Board (IRB00008635), ethical clearance number 140/Panke.KKE/II/2017.

## Conflict of Interest

The authors of this study declare that there was no conflict of interest.

## Acknowledgments

We would like to thank all the patients that participated in this study.
